# A Direct Comparison of Afferents to the Rat Anterior Thalamic Nuclei and Nucleus Reuniens: Overlapping But Different

**DOI:** 10.1523/ENEURO.0103-20.2021

**Published:** 2021-09-10

**Authors:** Mathias L. Mathiasen, Andrew J. D. Nelson, Eman Amin, Shane M. O’Mara, John P. Aggleton

**Affiliations:** 1 School of Psychology, Cardiff University, Cardiff CF10 3AT, United Kingdom; 2 Institute of Neuroscience, Trinity College Dublin, Dublin 2, Ireland

**Keywords:** frontal, hippocampus, layer 6, layer VI, subiculum, thalamus

## Abstract

Both nucleus reuniens and the anterior thalamic nuclei are densely interconnected with medial cortical and hippocampal areas, connections that reflect their respective contributions to learning and memory. To better appreciate their comparative roles, pairs of different retrograde tracers were placed in these two thalamic sites in adult rats. Both thalamic sites receive modest cortical inputs from layer V that contrasted with much denser projections from layer VI. Despite frequent overlap in layer VI, ventral prefrontal and anterior cingulate inputs to nucleus reuniens were concentrated in the deepest sublayer (VIb). Meanwhile, inputs to the anterior thalamic nuclei originated more evenly from both sublayers VIa and VIb, with the result that they were often located more superficially than the projections to nucleus reuniens. Again, while the many hippocampal (subiculum) neurons projecting to nucleus reuniens and the anterior thalamic nuclei were partially intermingled within the deep cellular parts of the subiculum, cells projecting to nucleus reuniens consistently tended to lie even deeper (i.e., immediately adjacent to the alveus). Variable numbers of double-labeled cells were present in those cortical and subicular portions where the two cell populations intermingled, though they remained in a minority. Our data also show how projections to these two thalamic sites are organized in opposing dorsal/ventral and rostral/caudal gradients across both the cortex and hippocampal formation. While the anterior thalamic nuclei are preferentially innervated by dorsal cortical sites, more ventral frontal sites preferentially reach nucleus reuniens. These anatomic differences may underpin the complementary cognitive functions of these two thalamic areas.

## Significance Statement

Both nucleus reuniens and the anterior thalamic nuclei link frontal cortical areas with the hippocampal formation. We show that the cortical and hippocampal projections to these thalamic sites show intermingled but opposing gradients of origin across the cerebral cortex (anterior thalamic, more dorsal; nucleus reuniens, more ventral), with their respective afferents typically arising from different neurons. There is also a repeated tendency across cortical areas for nucleus reuniens inputs to arise from the very deepest layer (VIb), while anterior thalamic inputs are often slightly more superficial, located across VIb and VIa. A similar depth distinction is again seen in the subiculum. These patterns indicate a separation of information reaching these two thalamic sites, alongside functional divisions within cortical layer VI.

## Introduction

Nucleus reuniens and the anterior thalamic nuclei (ATNs) both have dense, direct connections with the hippocampus and frontal cortices (Meibach and Siegel, 1977a,b; Herkenham, 1978; Shibata, 1993; McKenna and Vertes, 2004; Shibata and Naito, 2005; Vertes et al., 2007; Wright et al., 2013; Varela et al., 2014). The two thalamic areas also contain spatially responsive neurons (Taube, 1995, 2007; Tsanov et al., 2011; Jankowski et al., 2014, 2015; Matulewicz et al., 2019), with lesions in both sites disrupting spatial tasks that depend on the integrity of the hippocampus (Sutherland and Rodriguez, 1989; Warburton et al., 2001; Griffin, 2015; Clark and Harvey, 2016; Barker and Warburton, 2018; Viena et al., 2018). Despite these similarities, the two thalamic areas appear to have complementary functions (Aggleton et al., 2010; Cholvin et al., 2013, 2018; de Vasconcelos and Cassel, 2015; Clark and Harvey, 2016; Prasad et al., 2017; Mathiasen et al., 2020).

The rat nucleus reuniens receives dense cortical afferents from the prelimbic, anterior cingulate, dorsal peduncular, orbital, and infralimbic areas, while other afferents arise from the subiculum, postsubiculum, retrosplenial, secondary motor, insula, and perirhinal cortices (Herkenham, 1978; McKenna and Vertes, 2004; Varela et al., 2014; Mathiasen et al., 2019). The anterior thalamic nuclei receive projections from many of the same areas, with those from the anterior cingulate cortex, retrosplenial cortex, and subiculum being particularly dense (Meibach and Siegel, 1977b; Seki and Zyo, 1984; Van Groen and Wyss, 1992, 2003; Shibata, 1998; Shibata and Naito, 2005; Wright et al., 2013). Other cortical inputs to the anterior thalamic nuclei include those from the secondary motor cortex, the postsubiculum, presubiculum, and parasubiculum (van Groen and Wyss, 1990a,b; Shibata and Naito, 2005; Wright et al., 2013). Meanwhile, the anterior thalamic nuclei also receive particularly dense subcortical inputs from the mammillary bodies (Seki and Zyo, 1984; Takeuchi et al., 1985; Shibata, 1992). Projections from the mammillary bodies to nucleus reuniens have also been described (McKenna and Vertes, 2004), although these appear considerably lighter than those reaching the anterior thalamic nuclei.

There is considerable overlap in the sources of the inputs to nucleus reuniens and the anterior thalamic nuclei. Indeed, almost every site that innervates the anterior thalamic nuclei also appears to reach nucleus reuniens. However, current comparisons between the afferents to these two thalamic sites are indirect, relying on separate studies. The present analysis made direct comparisons by injecting different retrograde tracers into the two thalamic target areas in the same rat. The anterior thalamic injections largely targeted the anteromedial (AM) nucleus as it is the principal recipient of frontal inputs to the anterior thalamic nuclei (Shibata and Naito, 2005; Wright et al., 2013). The objective was to uncover any systematic differences in the respective inputs to nucleus reuniens and the anterior thalamic nuclei.

## Materials and Methods

### Ethical standards

All procedures were performed in accordance with the Cardiff University animal care animal care committee regulations and followed the UK Animals Act 1986 (Scientific Procedures).

### Nomenclature and anatomic borders

The ATNs principally comprise the AM, anteroventral (AV), and anterodorsal (AD) nuclei (Swanson, 1992). Although the laterodorsal thalamic nucleus shares many properties with the anterior thalamic nuclei, it lacks mammillary body inputs. For this reason, it is treated as distinct. In the rodent brain, a separate interanteromedial nucleus (IAM) is also recognized at the midline (Swanson, 1992; Paxinos and Watson, 2005).

Nucleus reuniens (RE) is also located on the midline. It is not uniform, as shown by how its afferents target different subareas within the nucleus (Herkenham, 1978; Van der Werf et al., 2002; McKenna and Vertes, 2004). The ventral margins of nucleus reuniens variously border the dorsal hypothalamus or the xiphoid and paraxiphoid (PaXi) thalamic nuclei (Paxinos and Watson, 2005). Except for its most rostral and most caudal levels, where dorsal nucleus reuniens borders the IAM and central medial (CM) nuclei, respectively, the dorsal border of nucleus reuniens is adjacent to the rhomboid nucleus (Rh). A nucleus peri-RE (pRE) has been identified adjacent to its more caudal, lateral borders (Van der Werf et al., 2002).

The terms “hippocampal formation” and “hippocampal” refer to the dentate gyrus, CA fields, and subiculum (Burwell and Witter, 2002). Meanwhile, the presubiculum, postsubiculum, parasubiculum, and entorhinal cortex all comprise parts of the parahippocampal region, along with the perirhinal and postrhinal cortices (Burwell and Witter, 2002). While some authorities regard the postsubiculum as part of the presubiculum (Witter, 2002), we treat it as distinct (Van Groen and Wyss, 1990b; Swanson, 1992). We subdivide the subiculum into its components: dorsal subiculum (dSUB); ventral subiculum (vSUB); and intermediate subiculum (iSUB), which is defined as the subicular portion positioned caudal to the caudal end of CA1 and dentate gyrus (Kishi et al., 2000; Bast et al., 2009). For purposes of clarification, the general term “cortex” excludes the hippocampal formation, although the latter is an allocortical region. Finally, the term “ventral prefrontal” cortices collectively refer to the prelimbic cortex (PL), infralimbic cortex (IL), and dorsal peduncular cortex (DP). The term “medial prefrontal cortex” (mPFC) refers to these same areas but also includes the anterior cingulate cortex (ACC; Preuss, 1995).

### Animals

The study principally involved 18 adult male Lister Hooded rats (weight, 291–320 g; Harlan/Envigo) with either dual injections involving both thalamic sites (14 rats) or with only a single injection in one of the two thalamic sites (4 rats). In some of the latter cases, an injection with a second tracer was located in either a different thalamic nucleus or the mammillary bodies, but these injection cases are not included in the analyses. Before surgery, all animals were housed in groups (of two to four animals) under a 12 h light/dark cycle, with sufficient food to ensure that their weight was always >85% of their free-feeding weight. Postsurgery, all animals were housed in groups (typically of three) with food and water available *ad libitum*.

### General surgical procedures

All surgeries took place under isoflurane anesthesia (isoflurane–oxygen mixture, 1.5–2.5%) with the rat positioned in a stereotaxic frame (Kopf Instruments). Stereotaxic coordinates were initially derived from a brain atlas (Paxinos and Watson, 2005) and were later refined. In all cases, the craniotomy was made on the right hemisphere, but an oblique syringe path (6°) helped to target nucleus reuniens at the midline. Lambda and bregma were set at the same depth coordinates, creating a flat skull.

As a part of the analgesia regime, lidocaine was administered topically to the scalp (0.1 ml of 20 mg/ml solution; B. Braun), and meloxicam was given subcutaneously (0.06 ml of 5 mg/ml solution; Boehringer Ingelheim).

### Retrograde tracer injections in nucleus reuniens and the anterior thalamic nuclei

In each of the 14 cases with dual injections, two retrograde tracer injections were made, one directed at nucleus reuniens, the other at the anterior thalamic nuclei (Table 1). The two tracers were Fast Blue (FB; Sigma-Aldrich), and cholera toxin b (CTB; List Biological Laboratories; 1% solution in 0.05 m Tris). These two retrograde tracers have previously been shown to effectively double label cell populations (Kinnavane et al., 2018). All tracers were injected either mechanically via a 0.5 or 1.0 μl Hamilton pipette or iontophoretically (all CTB injections except for cases #5 and #16). Mechanical tracer injections were infused with a flow of 20 nl/min, and the injection volume varied between 50 and 60 nl. In all cases, the needle was left in place for a further 10 min before being retracted. For iontophoretic injections, the injection time was 15 min using a current that varied among 2, 6, and 7 μA (5 min for each of the current settings). Finally, the same tracers and injection methods were used for the additional four cases in which just one of the target nuclei was involved.

**Table 1:** Tracer injection details for the study cases

Case #	CTB injection site	FB injection site	mPFC layer VI: main distribution of retrogradely labeled cells	mPFC layer V: (typically, many fewer cells than in VI)	Distribution of retrogradely labeled cells in the subiculum
#1 (216#10)	RE	AM/AV/AD	RE: layer VIb (+ scant label VIa) ATN: layer VIa + VIb	RE: only a few single scattered cells in layer V ATN: Layer V	RE: deep ATN: deep(↑)
#2 (5000#1)	AM	RE	RE: layer VIb (+ moderate VIa, predominantly in aACC) AM: layer VIa and VIb	RE: layer V AM: Layer V	RE: deep layer (+ scant in superficial layer) AM: deep layer(↑) (+ a few scattered cells in superficial layer)
#3 (223#10)	AM	RE	RE: layer VIb (moderate VIa in aACC) AM: layer VIa + VIb	RE: only a few single cells in layer V AM: Layer V	RE: deep (+ scant superficial cells only septal distal portion) AM: deep(↑) (a few single cells more superficial)
#4 (5000#2)	AM (VA)	RE (Rh)	RE+: layer VIb (+ scant in VIa) AM+: layer VIa and VIb	RE+: layer V AM: layer V	RE+: deep layer (+ scant in superficial layer) AM: deep layer(↑)
#5 (198#4)	AV	RE	RE: layer VIb (however, moderate- to-dense in VIa, mainly ACC) AM: layer VIa and VIb	RE: layer V moderate AM: layer V	RE: deep layer (+ scant in superficial layer) AM: deep layer(↑) (+ scant in superficial layer). In deep layer noticeably separate from deeper RE label
#6 (5000#7)	AM	RE (Rh/CM/SMT)	RE+: layer VIa and VIb (although densest in VIb) AM: layer VIa and VIb	RE+: layer V AM: layer V	RE+: deep layer (+ scant in superficial layer AM: deep layer(↑)
#7 (4000#3)	AM	RE (SMT, pRE)	RE+: layer VIb (moderate rostral ACC label in layer VIa) AM: layer VIa + VIb	RE+: layer V AM: layer V	RE+: deep (+ scant superficial label) AM: deep(↑) (a few single cells scattered superficially)
#8 (4000#7)	AM	RE/Rh	RE+: layer VIb (moderate rostral ACC label in layer VIa) AM: layer VIa + VIb	RE+: layer V AM: layer V	RE+: deep (+ scant superficial label) AM: deep(↑)
#9 (208#9)		RE	RE: layer VIb		RE: deep
#10 (215#4)		RE/Rh	RE+: layer VIb (rostral ACC moderate label in layer VIa)	N/A	RE+: deep (+ scant superficial label)
#11 (88#5)		AV	AV: layer VIa + VIb	AM: layer V	AM: deep (+ single scattered cells in superficial layer)
#12 (207#2)		RE/Rh/SMT (IAM)	RE+: layer VIa + VIb	RE+: layer V	RE+: deep (+ scant superficial layer)
#13 (4000#4)	AM	RE/Rh/CM, (pRE, SMT)	RE+: layer VIb + Via AM: layer VIa + VIb	RE+: layer V AM: layer V	RE+: deep (+ scant in superficial layer) AM: deep(↑)
#14 (5000#10)	AM/VM	RE	RE: layer VIb AM+: layer VIa and VIb	RE: a few single cells AM+: layer V	RE: deep layer AM+: deep layer(↑)
#15 (216#8)	Ventral RE/PaXi	AM/PT/MD/ VL/SMT	RE+: layer VIb (+ scant in layer VIa) ATN+: layer VIa + VIb	RE+: layer V ATN+: layer V	RE+: deep (moderate in superficial layer) ATN+: deep(↑)
#16 (198#8)	Ventral RE and PaXi (hyp, very weak involvement)	AM/MD (AV, AD)	RE+: layer VIb (+scant in layer VIa) ATN+: layer VIa + Vib	RE+: layer V ATN+: Layer V	RE+: deep + moderate superficial ATN+: deep(↑) (only single cells superficial)
#17 (216#12)	Ventral RE (PaXi, very minor involvement)	AM/MD (AV, AD, DG)	RE+: layer VIb (+ scant in layer Via) ATN+: layer VIa + VIb	RE+: layer V (dense in ventral mPFC) ATN+: layer V	RE+: deep + moderate superficial ATN+:(↑) deep (moderate superficial involvement in some sections)
#18 (216#3)	RE (very restricted)	AM/PC/MD/CM (AD, PT)	RE: layer VIb (+ scant in layer VIa) ATN+: layer VIa + VIb	RE: few scattered cells in layer V ATN+: layer V	RE: deep (only in iSUB where very scant) ATN+: deep(↑)

The top rows are those cases with the most selective pairs of injections. In column 1, the case numbers given in the text are in first line (#1 to #18), small case numbers in parenthesis (second line) give the original case numbers. In columns 2 and 3, areas in parentheses show only limited tracer. Column 4 indicates the overall distribution of retrograde cell labeling across the two sublayers of layer VI. Column 5 indicates whether the layer V label is present, while column 6 indicates the laminar position of label in the subiculum. In column 6, “deep” and “superficial” refer to the two principal cell layers. In cases with dual injections, (↑) indicates that the labeling is overall more superficial than the labeling from the other tracer, although still in the deep cell layer. DG, Dentate gyrus; FX, fornix; hyp, hypothalamus; MD, mediodorsal thalamic nucleus; PT, paratenial nucleus; SMT, submedial thalamic nucleus; VA, ventral anterior thalamic nucleus; VL, ventrolateral nucleus; PC, paracentral thalamic nucleus.

Compared with Fast Blue, the center of a CTB injection can occasionally be more difficult to define. Therefore, in four cases (#2, #4, #6, #8) we added the anterograde tracer biotinylated dextran amin (BDA; 10 kDa; Thermo Fisher Scientific) to the CTB tracer solution before iontophoretic infusion (1:1 BDA/CTB), using the same parameters as in the CTB injections. In these cases, staining for BDA should help reveal cellular uptake at the injection site and, thereby, reveal the core of the CTB injection site (the anterograde BDA transport was not analyzed).

### Histology and data analysis

After a survival time of 6–8 d, animals received a 1.5–2.0 ml intraperitoneal pentobarbital injection (Euthatal, Merial) and were transcardially perfused with 0.1 m PBS solution, immediately followed by paraformaldehyde (PFA) perfusion (4% PFA solution in 0.1 m PBS). The brains were postfixed for 4 h in the same PFA solution, then stored overnight in a 25% sucrose solution (25% in 0.1 m PBS). Sections were cut in the coronal plane with a freezing microtome (40 or 50 μm in four series or, in a single case, 20 μm in eight series). Two series from each case were initially studied. One series was directly mounted on gelatin-subbed slides (for Nissl stain) and another was placed in PBS at 4°C (for antibody immunohistochemistry).

The mounted sections were dried overnight, rehydrated in a series of ethanol solutions of decreasing concentrations (2× 100%, 90%, 70%), then stained with cresyl violet after 2 min in deionized water. Following cresyl violet staining, sections were again placed in deionized water, dehydrated (70%, 90%, 2× 100% ethanol series), defatted in xylene, and finally coverslipped with DPX (Thermo Fisher Scientific).

In contrast to Fast Blue, the tracer CTB requires immunohistochemical processing to be visualized. All immunohistochemical procedures were conducted at room temperature. For CTB staining, sections were washed for 3 × 10 min in 0.1 m PBS, washed 3× 10 min in PBS-TX (0.2% Triton X-100 in 0.1 m PBS), and incubated with the primary antibody rabbit anti-CTB overnight (1:3000; Sigma-Aldrich). Sections were washed for 3× 10 min in PBS-TX and incubated with the DyLight 594-conjugated secondary antibody goat anti-rabbit (1:200; Vector Laboratories) for 2 h. After a further 3× 10 min wash in PBS, sections were mounted on gelatin-subbed slides and dried overnight. Sections were then further dehydrated in ethanol (50%, 70%, 90%, 2× 100%), defatted in xylene, and coverslipped with DPX. In the four cases with 10 kDa BDA added to the CTB solution, the BDA tracer was visualized in an additional series of sections. In these sections, we stained with Alexa Fluor 488-conjugated streptavidin (1:200; Thermo Fisher Scientific) for 2 h (following standard procedures described above for CTB immunohistochemistry), followed by a cresyl violet stain. This method enabled us to accurately estimate the extent of the injection site.

In one anterior thalamic/reuniens case, we also stained an additional series for parvalbumin (case #3). Following the same overall protocol as for CTB fluorescence, we stained for anti-parvalbumin (1:10,000 dilution; Sigma-Aldrich) followed by the goat anti-mouse Alexa Fluor 488 antibody (Abcam). In this case, however, both the primary and secondary antibodies were incubated with a 1% NGS PBS-TX solution, and sections were washed for 90 min in a 5% NGS solution (in PBS-TX between incubation with the primary and the secondary antibodies). Two further cases (#9, #12) were also stained for parvalbumin, which both had single tracer injections in nucleus reuniens and in the mammillary bodies. The same visualization procedures were used except that the DyLight 594 secondary antibody (Vector Laboratories) was used in case #9.

### Handling of image data

A Leica DM5000B microscope with a Leica DFC310FX digital camera and Leica Application Suite image acquisition software was used for both bright-field and fluorescence microscopy. The latter involved the Leica fluorescence filter A4 (for Fast Blue label), N21 (for CTB label), and L5 (for green background only). Fluorescence photomicrographs acquired for illustration purposes were occasionally adjusted for contrast, brightness, and intensity.

Overlays were made (Corel Photo-Pain X8) from images of cresyl violet-stained sections alongside images of the corresponding fluorescence sections (with retrograde-labeled cells and/or parvalbumin stain). To establish the precise position of the thalamic injections and the laminar distribution of retrograde-labeled cells, in selected sections we additionally imaged Fast Blue- and BDA-labeled cells in sections stained with cresyl violet.

### Statistical analysis

In seven cases with selective dual injections, we counted labeled cell numbers in the medial prefrontal cortex, retrosplenial cortex and subiculum, noting those double-labeled cells (see “Results” for case numbers). We counted cells along the anteroposterior and dorsoventral extent of the regions, occasionally avoiding retrosplenial sections very close to the syringe tract.

Labeled cells were manually counted with the aid of Olympus CellSense, Inkscape, and the CorelDRAW Graphics Suite software.

The total number of labeled cells in a given region was divided by the number of counted sections. For the subiculum cell count, two cases were excluded from the statistical analysis as either the labeling signal in ventral subiculum was markedly attenuated (case #3) or the subiculum cell numbers constituted a clear outlier compared with the remaining dataset (+2 SDs below the mean; case #1). While cells in both hemispheres were counted (see Mathiasen et al., 2017), only those counts in the hemisphere ipsilateral to the anterior thalamic nucleus injection were included in the statistical analyses.

Both SPPS (statistics 25) and JASP (0.14.1.00) software were used for the data analyses. As the SD for the cell counts was proportional to the mean (minimum *r* = 0.8, *p* = 0.015), all data, including the sublayer cell counts, were subject to logarithmic transformations (Howell, 2002). To analyze the raw cell counts, a within-subject ANOVA with factors of cortical region (anterior ACC, posterior ACC, PL, IL/DP), subicular subregion (dorsal, intermediate, ventral), or retrosplenial cortex as well as injection site (ATN vs RE) was conducted on the transformed data. The raw counts are expressed relative to the size of the region of interest (in square millimeters). The region of interest comprised layer VI in cortical regions as well as the deep layer in subiculum. This approach allowed us to compare cell numbers in different portions of subiculum, independently of size variations caused by the coronal cutting plane.

To assess whether there were topographical differences in labeling within cortical layers VI, a proportion was calculated by dividing the cell counts in layer VIa by the total cell counts in layer VIa and VIb. These proportions were calculated separately for the two injection sites. These data were then analyzed by within-subject ANOVA with factors of injection site (RE, ATN) and region (prelimbic cortex, anterior ACC, posterior ACC).

Finally, to assess whether ATN or RE inputs make up a higher proportion of double-labeled cells in a site, a proportion was calculated by dividing the number of double-labeled cells in a given region by the total number of cells in that region. To examine whether there were regional differences in the number of double-labeled cells, a one-way ANOVA with factor of cortical region (anterior ACC, posterior ACC, PL, IL/DP) or subicular subregion (dorsal, intermediate, ventral) was also performed. Statistical comparisons were not, however, made comparing the RE and ATN cell counts as the data are not independent (the double-labeled cells are shared).

A simple effects analysis (based on the pooled error term) was used to explore significant interactions. *Post hoc* tests (Bonferroni) were used to examine significant simple main effects or significant main effects. For all analyses, the α level was *p *<* *0.05 for the rejection of the null hypothesis.

In the three parvalbumin-stained cases, we measured the parvalbumin signal intensity in ImageJ. From these three brains, a total of 22 sections was analyzed, including all frontal regions. For each section, two regions of interest (ROIs) were drawn. One delineating layer VIb, and another layers II-VIa combined. The average pixel intensity in each ROI were measured. A two-tailed *t* test examined signal differences between these layers.

### Data availability

All cases described are imaged, and these, as well as the quantitative dataset, are available on request to the communicating author.

## Results

Both the cortical and subcortical inputs to the anterior thalamic nuclei and nucleus reuniens have separately been described in considerable detail (Herkenham, 1978; Seki and Zyo, 1984; Shibata, 1992; McKenna and Vertes, 2004; Shibata and Naito, 2005; Wright et al., 2013). Consequently, the emphasis is on comparisons between the two sets of afferents and not to repeat that already described.

### Injection sites

**Figure 1.**
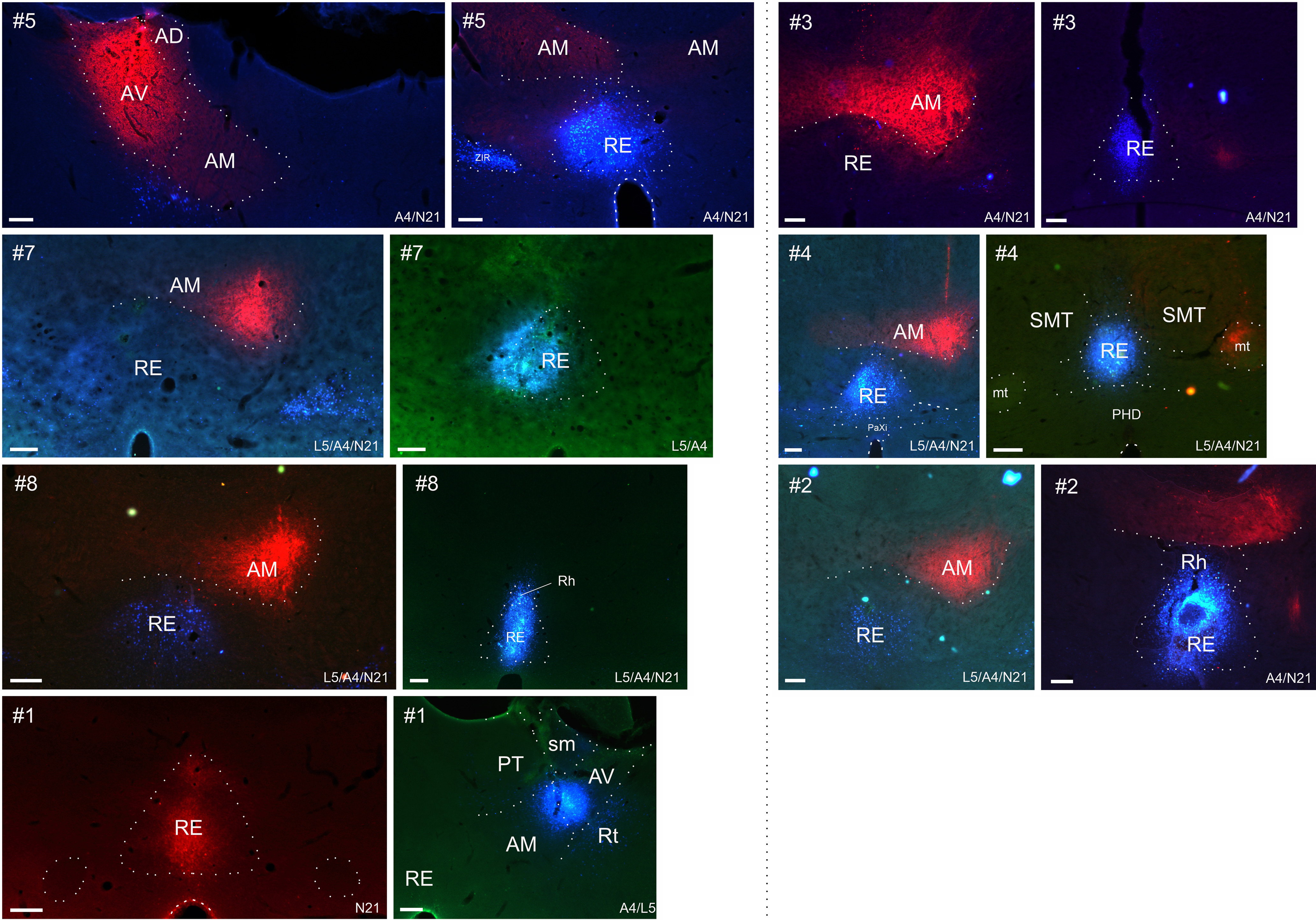
Photomicrographs showing the CTB and FB tracer deposits in the seven highlighted cases. Case numbers are indicated in the top left corner. In all cases, the red tracer is CTB. Images show the center of each injection. L5/A4/N21 refers to the filter cubes used for imaging in the epifluorescence microscope. The dotted lines signal nuclei boundaries. AM, anteromedial thalamic nucleus; AV, anteroventral thalamic nucleus; mt, mammillothalamic tract; PHD, posterior hypothalamic area, dorsal part; PT, parataenial nucleus; RE, nucleus reuniens; Rh, rhomboid thalamic nucleus; Rt, reticular nucleus; sm, striamedullaris; SMT, submedial thalamic nucleus; ZIR, zona incerta rostral part. Scale bars, 200 μm.

The results principally come from 14 animals in which one tracer was centered within nucleus reuniens, the other within the anterior thalamic nuclei (Fig. 1, Table 1). In eight of these cases (#1, #2, #3, #5, #6, #7, #8, and #13) the tracer injection in the anterior thalamic nuclei appeared completely restricted to these nuclei. In the remaining cases, the injections were centered in the anterior thalamic nuclei, but the borders of some adjacent structures (but not nucleus reuniens) were reached.

Meanwhile, the tracer injections in reuniens appeared either completely confined to that nucleus (cases #1, #2, #3, #5, #14, and #18), had very limited involvement of other ventral midline structures (cases #4, #6, #7, and #17), or appeared to have more extensive involvement of the ventral midline thalamus or dorsal hypothalamus (cases #8, #13, #15, and #16).

Of the additional four cases (i.e., those with only a single injection), the Fast Blue injection in case #9 appeared confined to nucleus reuniens while that in case #11 appeared confined to the anterior thalamic nuclei (Table 1). Meanwhile, cases #10 and #12 are of interest as the Fast Blue injection appeared confined to the thalamic midline, principally involving nucleus reuniens and the rhomboid nucleus. These four cases help to confirm the laminar and regional projection patterns seen in the cases with dual injections.

Seven cases with discrete injections involving both nucleus reuniens and anterior thalamic nuclei are particularly highlighted, these same cases being used for the quantitative analysis. In four of these cases, both injections were confined within nucleus reuniens and the anterior thalamic nuclei (cases #1, #2, #3, and #5). In two cases (#7 and #8), one injection was confined in the anterior thalamic nuclei, but the nucleus reuniens tracer protruded into the perireuniens and submedius thalamic nuclei (case #7) or the rhomboid nucleus (case #8). Finally, in case #4, the anterior thalamic injection just reached the ventral anterior nucleus, while the injection in reuniens reached into the immediately adjacent rhomboid nucleus.

In these seven cases, the anterior thalamic injection was positioned in AM in five cases (cases #2, #3, #4, #7, and #8) and AV in one case (case #5), and included all three nuclei (AM/AV/AD) in the final case (#1). For details of the remaining cases, see Table 1.

### Cortical layer VI sublayers

It became evident that the location of projections from within layer VI differed for the two thalamic sites. For this reason, we begin by distinguishing two sublayers within layer VI.

#### Anterior cingulate cortex and secondary motor cortex

**Figure 2.**
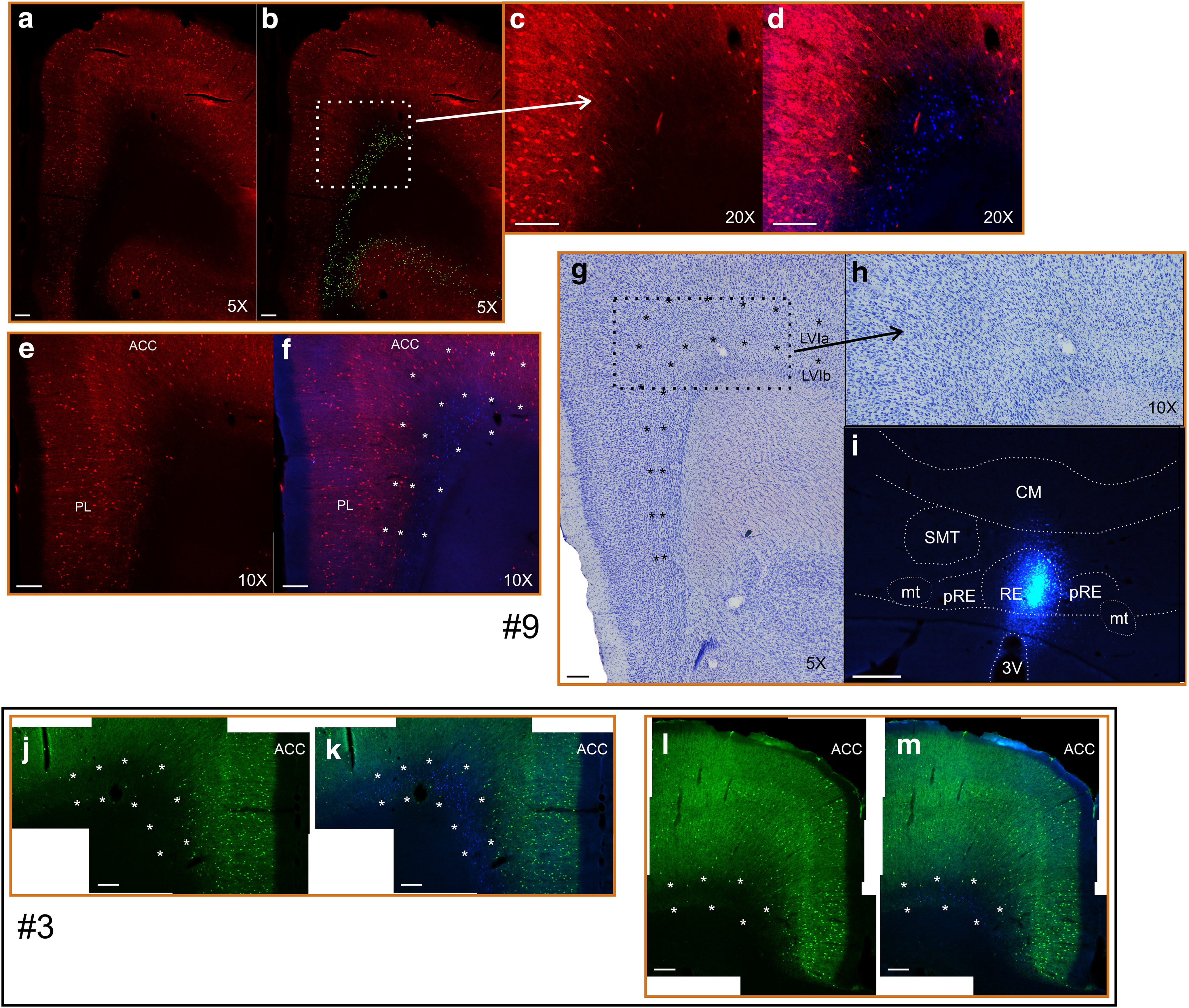
Photomicrographs depicting the delineation of sublayers VIa and VIb in cresyl violet- and parvalbumin-stained sections. ***a–h***, ***j–m***, The images also show how retrogradely labeled cells from Fast Blue in nucleus reuniens are preferentially localized in layer VIb in cases #9 (***a–h***) and #3 (***j–m***). Notice that the parvalbumin stain is stained with a red fluorophore in case #9 and green in case #3. ***a***, ***b***, A parvalbumin-stained frontal section (red) with Fast Blue-labeled cells plotted (green/yellow color) in ***b***. ***c***, ***d***, A 20× zoom scan of the frontal cortical portion indicated by the white stippled box in ***b***. ***c*** shows the parvalbumin label, while ***d*** shows both parvalbumin and Fast Blue labels. ***e***, ***f***, Frontal brain sections as in ***a*** and ***b***, but with the sublaminar differentiation of layer VI indicated in ***f***. Inserted asterisks indicate the borders between layers VIb/VIa and layers VIa/V, as well as the cingulum bundle/layer VIb border. ***g***, The laminar delineations are based on cytoarchitectonic criteria. Nissl-stained section from the same case (#9) is shown, with the sublaminar borders indicated by asterisks as in ***f***. This brain section is adjacent to the section in ***e*** and ***f***, and the indicated laminar borders in this case were made by overlaying the image with the cresyl violet-stained image. ***h***, Enlargement of the portion indicated in ***g*** with a black stippled box. ***i***, Center of the injection site in case #9. ***j–m***, Images from case #3, showing parvalbumin (green) with Fast Blue (***k***, ***m***) or without Fast Blue (***j***, ***l***) in rostral anterior cingulate/prelimbic cortices (***j***, ***k***) and a more caudal portion of the anterior cingulate cortex (***l***, ***m***). Inserted asterisks indicate the border between layers VIa/VIb and VIb/cingulum bundle. Note that retrogradely labeled cells in ***b*** are manually plotted whereas in all other images the original scans are shown. Images are adjusted for contrast, brightness, and intensity. 3V, Third ventricle; mt, mammillothalamic tract; SMT, submedial thalamic nucleus; ACC, anterior cingulate cortex; RE, nucleus reuniens; CM, central medial thalamic nucleus; pRE, perireuniens. Scale bars, 200 μm.

In keeping with many authorities (Paxinos et al., 1999; Jones et al., 2005; Vogt and Paxinos, 2014), layer VI can be subdivided into at least layers VIa (superficial) and VIb (deep). The two sublayers are particularly distinct in cortical areas lateral to the anterior cingulate cortex, including the secondary motor cortex. Here, an obvious cell-sparse zone differentiates layer VI into two portions, where the deepest portion comprises a rather narrow band of darkly Nissl-stained cells (Fig. 2*g*,*h*). In our terminology, layer VIb includes both the cell-sparse zone and the band of darkly stained cells (but see Paxinos et al., 1999). In the anterior cingulate cortex, the cell-sparse zone is only occasionally present (mainly at its lateral portions), although the cytoarchitectonic differences between layers VIa and VIb persist.

In addition to these cytoarchitectonic criteria, we observed that the intensity of cell and neuropil parvalbumin label differentiated these sublayers (Fig. 2). In the secondary motor cortex, the parvalbumin signal in the cell-sparse zone of layer VIb is extremely weak, with diminished labeling at the deepest portion. In contrast, layer VIa is more clearly labeled. In the anterior cingulate cortex, layer VIb is virtually devoid of parvalbumin label, which, again, contrasts with a clear signal on layer VIa. This signal in layer VIa is, however, weaker than that seen in layer V.

#### Ventral prefrontal cortices

In prelimbic cortex and infralimbic cortex, the deep sublaminar differentiation (based on cytoarchitecture) was again present. As in the anterior cingulate cortex, layer VIb is virtually devoid of parvalbumin labeling, while layer VIa contains obvious parvalbumin labeling. The border between layers VIa and layer V is, however, indistinct in parvalbumin-stained sections. Layer VIa appears narrower in the ventral prelimbic and infralimbic cortices than in more dorsal cortical areas. Likewise, in the dorsal peduncular cortex, the deep cell layer is differentiated by a lack of (or extremely diminished) parvalbumin stain and appears continuous with the infralimbic layer VIb (also observed in cresyl violet-stained sections). In lateral frontal areas, like the lateral orbital cortex (LO), neither parvalbumin stain nor cytoarchitecture differentiated layer VI sublayers (parvalbumin-labeled layer VI).

Combining sections from both anterior cingulate and ventral prefrontal cortices, the parvalbumin signal intensity in layer VIb (mean ± SEM, 6.34 ± 1.15) was significantly reduced compared with the signal intensity of other cellular layers (mean ± SEM, 18.60 ± 3.72; *t*_(2)_ = 4.57, *p* < 0.05).

### Distribution of cortical projections

**Figure 3.**
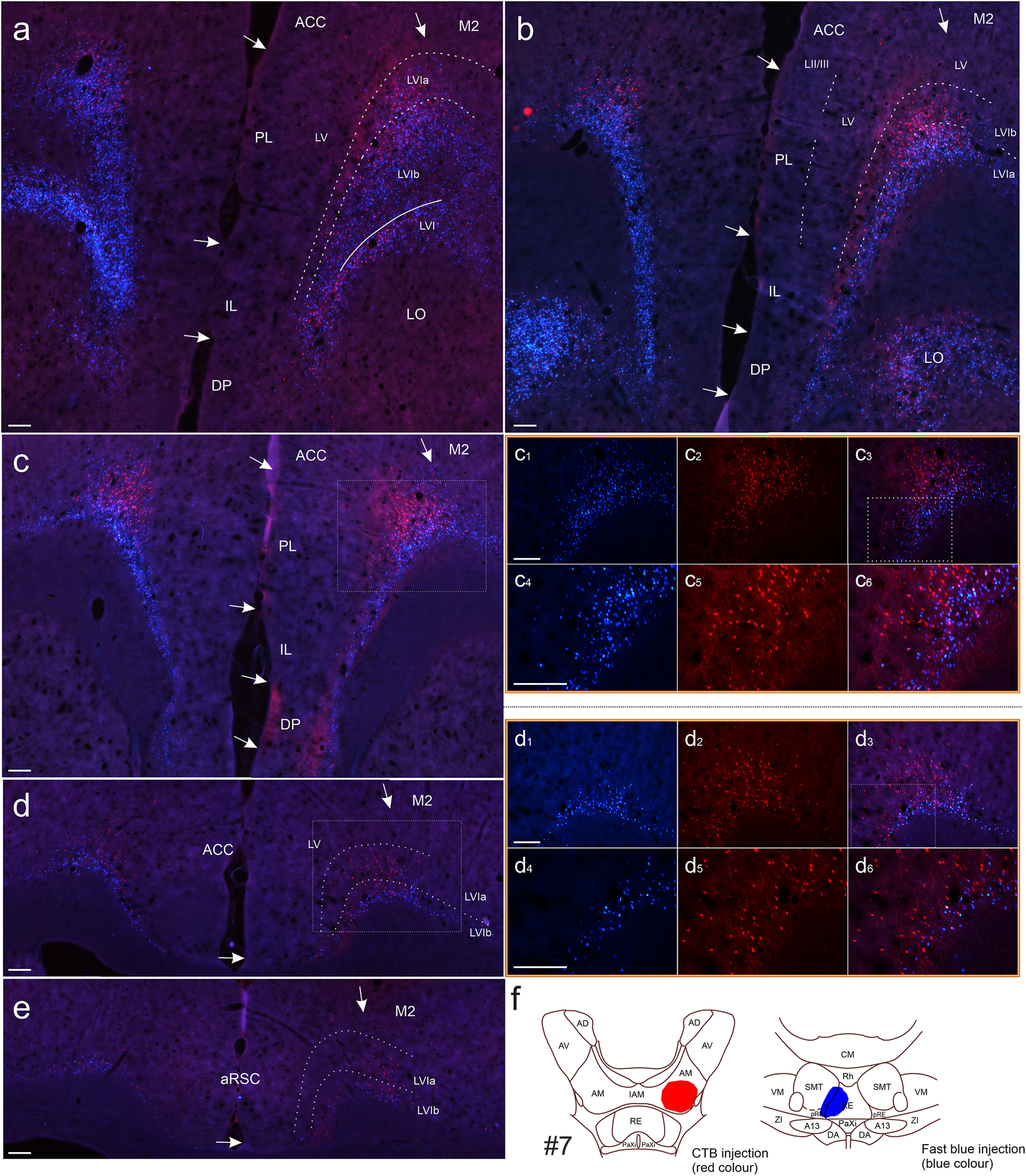
Photomicrographs showing the distribution of labeled cells resulting from a CTB injection in the anteromedial thalamic nucleus (red cells) and a Fast Blue injection in the nucleus reuniens (blue cells; case #7). ***a–e***, Scans (5×) illustrating cell labeling along the anteroposterior axis showing all portions of neocortex substantially labeled by both tracers. Stippled white lines indicate laminar borders, and arrows indicate regional borders. Cells labeled from the Fast Blue tracer in nucleus reuniens are densely concentrated in layer VIb in the ACC, PL, IL, and secondary motor cortices (M2), as well as the deep cell layer of the DP. At anterior portions of the anterior cingulate cortex only, appreciable Fast Blue labeling is also present in layer VIa. Cells labeled by CTB (anteromedial nucleus injection) were more concentrated in the anterior cingulate cortex and distributed in both layer VIa and layer VIb, but in some sections had a clear preference for layer VIa, avoiding the deepest portion of layer VIb. Some Fast Blue- and CTB-labeled cells are also present in layer V. In this case, cell labeling by both CTB and Fast Blue extends into the rostral retrosplenial cortex. ***c_1_*–*c_3_***, Zoom images (10×) of the box in ***c***. ***c_4_–c_6_***, Zoom images (20×) of the box in ***c_3_***. ***d_1_–d_3_***, Zoom images (10×) of the box in ***d***. ***d_4_–d_6_***, Zoom images (20×) of the box in ***d_3_***. In all zoom panels, the first image to the left shows Fast Blue-labeled cells, the second shows CTB-labeled cells, and the third image is an overlay of the two previous images. Double-labeled cells are present and can clearly be seen in the 20× zoom images. ***f***, Line drawing of the injection site. For photomicrographs of injections, see Figure 1. Images are adjusted for contrast, brightness, and intensity. A13, A13 dopamine cells; ACC, anterior cingulate cortex; AD, anterodorsal thalamic nucleus; AM, anteromedial thalamic nucleus; AV, anteroventral thalamic nucleus; CM, central medial thalamic nucleus; DA, dorsal hypothalamic areas aRSC, anterior retrosplenial cortex; DP, dorsal peduncular cortex; IL, infralimbic cortex; IAM, interanteromedial thalamic nucleus; LO, lateral orbitofrontal cortex; M2, secondary motor cortex; PaXi, paraxiphoid thalamic nucleus; PL, prelimbic cortex; pRE, perireuniens; RE, nucleus reuniens; Rh, rhomboid nucleus; SMT, submedial thalamic nucleus; VM, ventromedial thalamic nucleus; ZI, zona incerta. Scale bars, 200 μm.

We first provide an overall description, with Figure 3 depicting a representative case. Other cases then help to highlight some more precise aspects of the cell labeling. These descriptions are supported by quantitative analyses based on labeled cell counts from the seven selected cases (Fig. 1).

#### Anterior cingulate and secondary motor cortex

All cases with RE tracer injections contained frequent labeling in layer VIb of both the anterior cingulate and adjacent parts of the secondary motor cortex, including those cases where the injection was most restricted to RE (Fig. 3). In contrast, the corresponding retrograde label in layer VIa was sparser and more scattered, especially in the secondary motor cortex (though denser in those cases with some central medial thalamic involvement). At rostral anterior cingulate levels (aACC), the RE cell plexus extended to give more moderate labeling in layer VIa (though this label tended to be more lightly scattered for those cases with restricted reuniens injections). At more caudal anterior cingulate levels (pACC), the RE cell labeling was typically more restricted to layer VIb (Fig. 3*d*).

These sublayer distributions were verified in two cases with Fast Blue injections restricted to RE and additionally stained for parvalbumin (Fig. 2*b*,*d*,*f*,*k*,*m*, cases #3, #9). In both cases, the Fast Blue labeled cells were concentrated in the deep laminar portions that were largely devoid of parvalbumin stain (i.e., layer VIb).

The ATN injections revealed a different pattern of layer VI labeling in the anterior cingulate and secondary motor areas (Fig. 3). Here, labeling in the secondary motor cortex was either absent or scattered in both layers VIa and VIb. In anterior cingulate cortex, labeled cells were likewise typically present in both layer VI sublayers (Fig. 3*a–d*). In many sections, the ATN cell label was clearly positioned superficially compared with the RE label, although there were also large portions where the cell populations were intermingled, and in these portions double-labeled cells were typically present. The two layer VI cell populations (RE from ATN) were typically more distinct at more caudal anterior cingulate levels.

In most cases, some additional cell labeling was present in layer V (Table 1) after injections involving both RE and ATN. There was, however, a particular lightness of the layer V label in those cases when the tracer appeared completely restricted to RE.

#### Ventral prefrontal cortices

**Figure 4.**
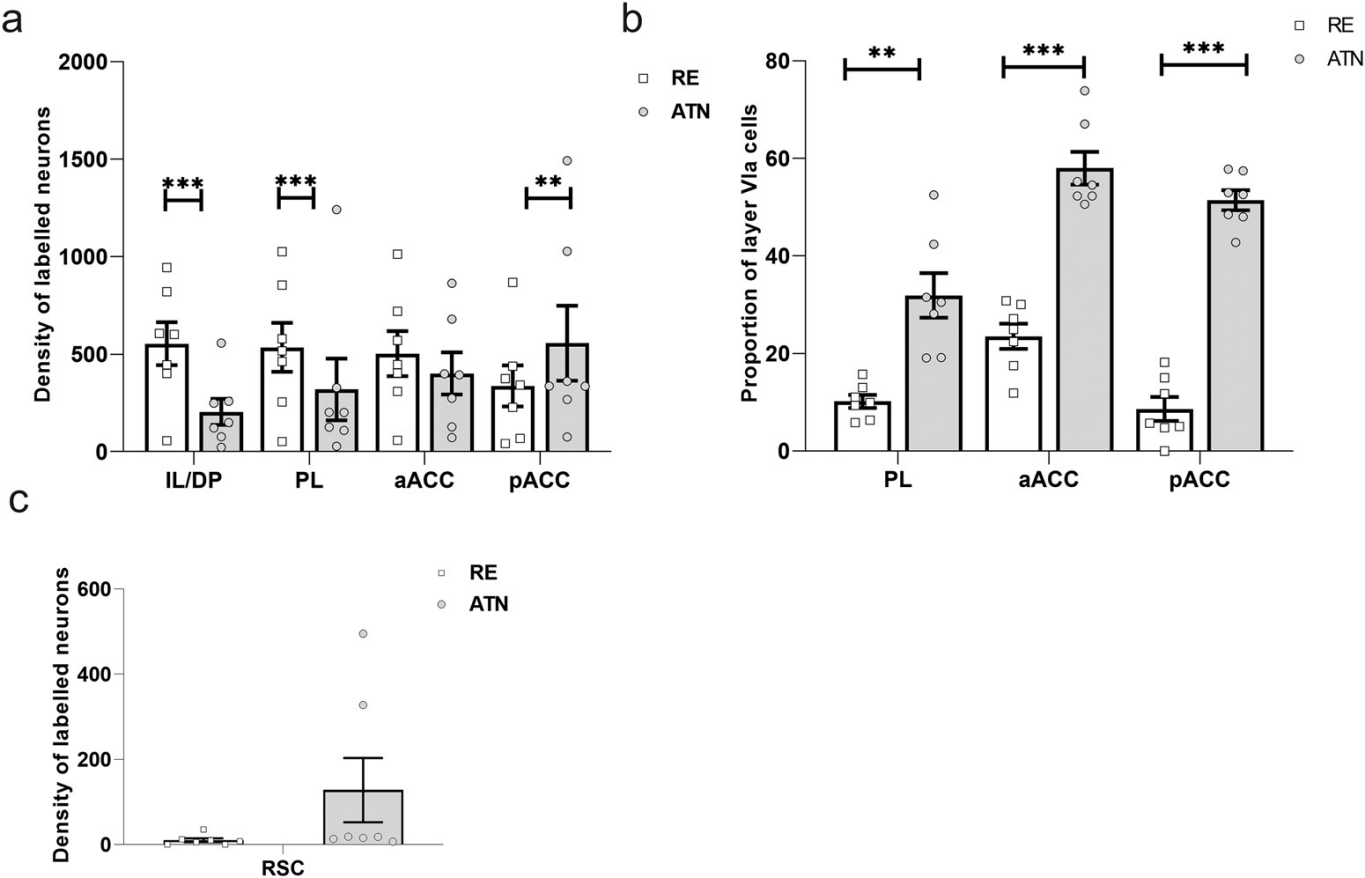
Density of cortical labeled neurons (per square millimeter): the plots show the mean and SEM from the seven highlighted cases. ***a***, Density of retrogradely labeled cells. Densities are given for the IL/DP, PL, as well as the aACC and pACC. ***b***, Cortical cell counts showing the number of cells in layer VIa as a proportion of the total cell counts in layer VIa and VIb combined. Cell counts are given for the same areas as in ***a***, except for IL/DP. ***c***, Density of retrogradely labeled cells in retrosplenial cortex (RSC). ATN, Anterior thalamic injections; RE, nucleus reuniens injections. ***p < 0.001; **p < 0.01.

In general, RE tracer injections labeled layer VIb in medial cortical areas, with noticeably weaker labeling in layer VIa (Figs. 3, 4*b*, Table 1). Labeled cells were often present along the entire dorsoventral extent of the medial prefrontal cortex; that is, in the anterior cingulate, prelimbic, and infralimbic cortices, and in the dorsal peduncular cortex. In the prelimbic and infralimbic cortices, the dense labeling in layer VIb typically ceased at the border with layer VIa, leaving much more scattered cell labels in the latter sublayer. The few exceptions, which showed a less abrupt VIa/VIb change of label density in these areas, had greater involvement of the paraxiphoid and/or hypothalamus in the RE injection site (Table 1).

The frontal labeling pattern in the 10 cases with the most restricted ATN injections was strikingly different. While appreciable labeling was present in the anterior cingulate and prelimbic cortex (primarily dorsal portion), much sparser label was seen in more ventral portions of the frontal cortex (dorsal peduncular cortex, infralimbic, and, in some cases, ventral prelimbic cortex). The prelimbic label was distributed more evenly across both layers VIa and VIb (Fig. 4*b*), thereby contrasting with the more confined layer VIb label after RE injections. Within this general pattern, following ATN injections there was an apparent overall preference for layer VIa at dorsal portions of prelimbic cortex, but labeling was either more equally scattered or more evident in layer VIb at more ventral portions.

Again, as in the anterior cingulate cortex, a lighter label in layer V was typically present in ventral prefrontal cortices following both ATN and RE injections. However, in most cases with injections restricted to RE, this label was either absent or particularly sparse.

Label was also present in layer VI of the lateral orbital field in most cases, including those with injections restricted to RE and ATN. The labeled cells were typically intermixed across this lamina, with no apparent difference in laminar depth.

#### Retrosplenial cortex

**Figure 5.**
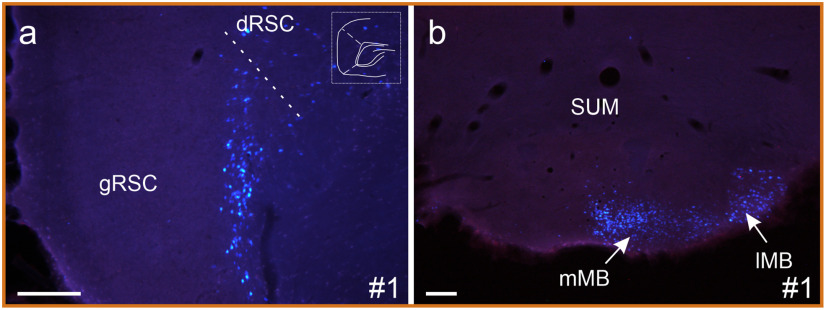
Photomicrographs showing retrograde cell labeling in case #1 with a Fast Blue injection involving all anterior thalamic nuclei combined with a CTB injection in nucleus reuniens. ***a***, Plentiful Fast Blue labeling in layer VI of retrosplenial cortex following the ATN injection, while no CTB-labeled cells are visible. The schematic drawing in the top right corner indicates a–p levels of the image. ***b***, Retrograde cell labeling in the mammillary bodies where only Fast Blue (ATN injection) is visible, with no cells labeled following the nucleus reuniens injection. lMB, Lateral mammillary nucleus; mMB medial mammillary nuclei; dRSC, dysgranular retrosplenial cortex; gRSC, granular retrosplenial cortex; SUM, supramammillary nucleus. Scale bars, 200 μm.

Starting with case #1, where the tracer injection involved multiple anterior thalamic nuclei, considerable labeling was consistently present, primarily in layer VI of the granular retrosplenial cortex (Fig. 5*a*). The retrosplenial labeling was also dense in cases with more restricted AV injections. However, in those cases with restricted AM injections, much lighter retrosplenial labeling was present (layer VI).

In contrast, in those injections seemingly restricted to RE, the retrosplenial cortex label was either absent or extremely sparse. More consistent retrosplenial labeling was observed in those cases (e.g., #7 and #12) where the RE injection involved other midline nuclei (Fig. 3*e*). This retrosplenial labeling was observed in layer VI of both granular and dysgranular divisions.

### Quantitative analyses

As described, all targeted cortical regions contained labels from both thalamic injection sites, but there were area differences (Fig. 4*a*).

ANOVA yielded no main effect of injection site (*F* < 1) or a main effect of region (*F*_(3,18)_ = 1.51, *p* = 0.246), but did show an interaction between injection site and region (*F*_(3,18)_ = 16.7, *p* < 0.001).

A simple effects analysis of this interaction revealed that there were higher cell counts in both prelimbic (*F*_(1,18)_ = 20.37, *p* < 0.001) and infralimbic/dorsal peduncular (*F*_(1,18)_ = 40.43, *p* < 0.001) following the RE injections relative to the counts in these regions following ATN injections. In the pACC, there were higher counts after ATN injections relative to RE injections (*F*_(1,18)_ = 8.94, *p* = 0.008), but there were no statistically reliable differences in cell counts between the two injections sites in the aACC (maximum *F*_(1,18)_ = 2.028, *p* = 0.171).

Furthermore, a simple effects analysis of the pattern of cell counts across the four regions following injections in ATN also revealed significant differences (*F*_(3,18)_ = 13.69, *p* < 0.001). *Post hoc* tests (Bonferonni corrected) confirmed that following ATN injections there were lower counts in both infralimbic/dorsal peduncular and prelimbic cortices relative to the caudal portion of anterior cingulate (minimum *p* = 0.007). There was also a simple main effect of region following RE injections (*F*_(3,18)_ = 5.57, *p* = 0.007), but *post hoc* tests found no statistically reliable differences between the four regions (minimum *p* = 0.11).

For the retrosplenial cortex (not included in the analysis above) cell numbers varied drastically depending on whether AV was involved in the ATN injection site. ANOVA therefore revealed only a marginal effect of injection site (*F*_(1,6)_ = 5.65, *p* = 0.055; Fig. 4*c*) but with an overwhelming abundance of ATN-projecting cells in the two cases with AV involvement in the injection (Fig. 4*c*, two outliers).

#### Laminar disposition of retrogradely labeled cells in cortical layer VI: VIa versus VIb

There was a repeated impression that the layer VI inputs to RE and ATN showed a partial segregation. In the three cortical areas examined, the RE inputs predominantly arose from the deepest layer (VIb). This impression was confirmed by examining the percentages of layer VIa cells (as a proportion of all labeled cells in layer VI) in the prelimbic cortex, and the rostral and caudal anterior cingulate cortices (Fig. 4*b*). Infralimbic/dorsal peduncular cortices were not included as a deep laminar differentiation was not observed in the dorsal peduncular cortex, and because of the occasional shrinking of layer VIa in the infralimbic cortex.

There was a main effect of injection site across all three target regions reflecting the overall higher proportion of RE projections from layer VIb relative to the projections to ATN (*F*_(1,6)_ = 116.69, *p* < 0.001). There was also a main effect of region (*F*_(2,12)_ = 23.82, *p* < 0.001) as well as a region by injection site interaction (*F*_(2,12)_ = 8.59, *p* = 0.005), reflecting how the degree of this laminar separation effect varied between cortical areas (Fig. 4*b*).

A simple effects analysis of this region by injection site interaction confirmed that in all three sites the proportion of ATN projections from layer VIa was higher than the RE projections from VIa (minimum *F*_(1,6)_ = 16.84, *p* = 0.006). Further simple effects analysis revealed an effect of region after RE injections (*F*_(2,12)_ = 8.18, *p* = 0.006), as the proportion of layer VIa cells was higher in rostral anterior cingulate relative to both prelimbic and caudal anterior cingulate cortices (*p* = 0.016). There was also an effect of region after ATN injections (*F*_(2,12)_ = 22.54, *p* < 0.001) as the ATN projections from prelimbic layer VIa comprised a lower relative proportion than that seen in both the rostral (*p* = 0.003) and caudal (*p* = 0.03) anterior cingulate cortices (Fig. 4*b*).

### Hippocampal formation: laminar, septotemporal, and dorsoventral organization of subiculum afferents

Cytoarchitectonic differentiation is visible within the cell layers of the subiculum. At septal levels, a principal cell layer, mainly consisting of large pyramidal cells, can be distinguished from a deep, more heterogeneous, narrow polymorphic cell layer. Both nonpyramidal and pyramidal cells are intermingled in this deep polymorph layer. In contrast to the neocortex (see above), parvalbumin staining does not differentiate between the subiculum cell layers.

Consistent with previous studies, the subiculum label following ATN injections showed a proximal–distal organization (e.g., anteromedial injections resulted in a more proximal label). Meanwhile, RE tracer injections led to cell labeling across the proximal–distal axis, occasionally with a preference for distal portions at septal levels.

**Figure 6.**
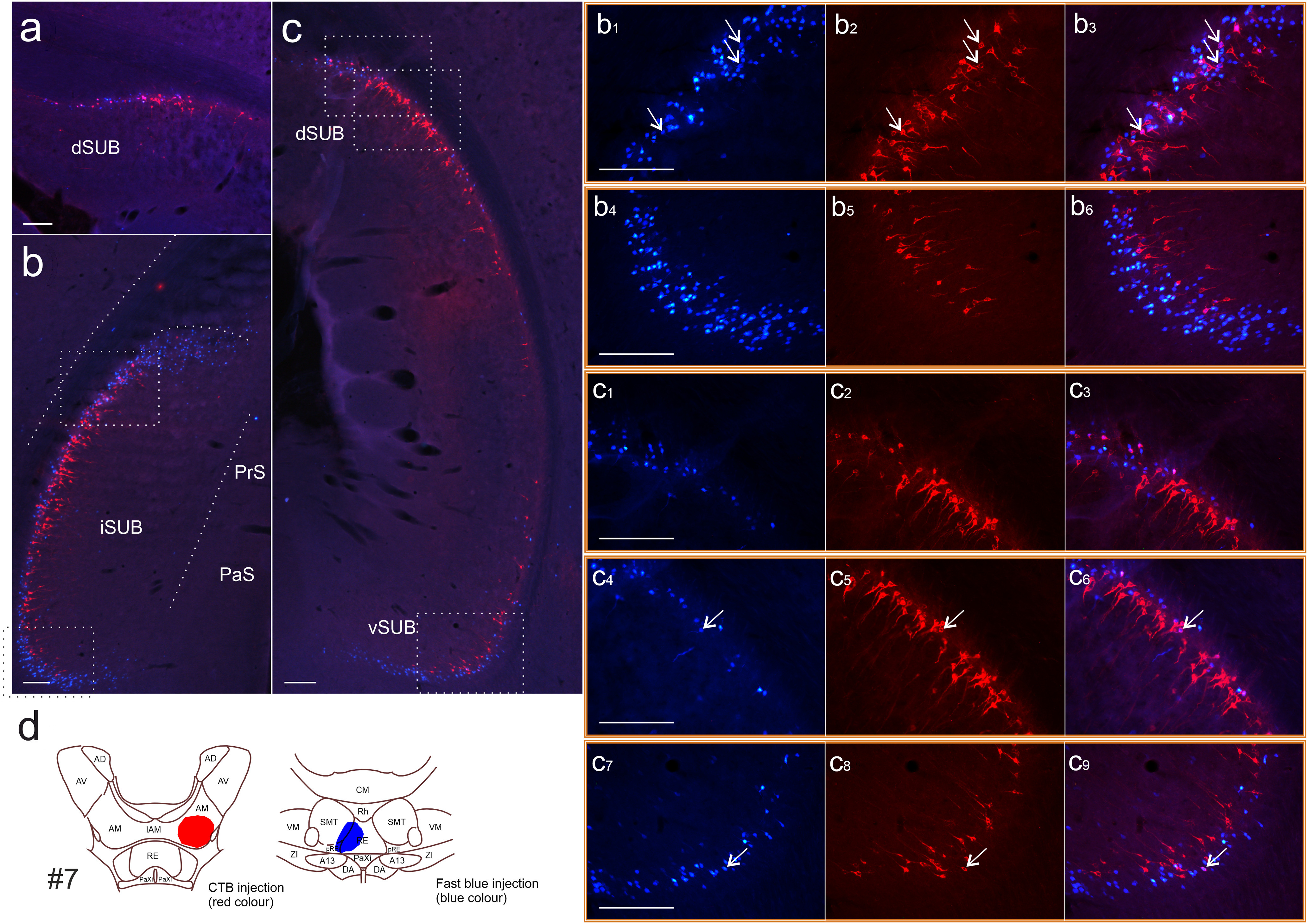
Photomicrographs depicting the distribution of label resulting from a CTB injection in the AM (red cells) and a Fast Blue injection centered in RE (case #7). ***a–c***, A 5× scan of cell labeling in subiculum. Labeled cells resulting from the Fast Blue tracer in nucleus reuniens are distributed across all septotemporal and proximodistal portions (with a slight preference for distal portions at septal levels), whereas the CTB label (from the anteromedial thalamic nucleus) shows a clear preference for the proximal subiculum and often avoids the most ventral subiculum, aside from the ventral portion of the intermediate subiculum (***c***). ***b_1_–b_3_***, Zoom images (20×) of the dorsal white box in ***b***. ***b_4_–b_6_***, Zoom images (20×) of the black/white ventral box in ***b***. ***c_1_*–*c_3_***, Zoom images (20×) of the most dorsal white box in ***c***. ***c_4_*–*c_6_***, Zoom images (20×) of the slightly more ventral white box in ***c***. Comparing ***c_1_–c_3_*** and ***c_4_–c_6_***, the proximodistal differences between the two cell populations are evident. ***c_7_–c_9_***, Zoom images (20×) of the most ventral white box in ***c***. Double-labeled cells are visible in the 20× zoom images, highlighted by arrows pointing to representative double-labeled cells. In all zoom panels, the first image shows Fast Blue-labeled cells, the second image shows CTB-labeled cells, and the third image is an overlay of the two. Images are adjusted for contrast, brightness, and intensity. ***d***, Line drawings depicting the center of the two injections in case #7. A13, A13 dopamine cells; AD, anterodorsal thalamic nucleus; AM, anteromedial thalamic nucleus; AV, anteroventral thalamic nucleus; CM, central medial thalamic nucleus; dSUB, dorsal subiculum; IAM, interanteromedial thalamic nucleus; iSUB, intermediate subiculum; PaS, parasubiculum; PaXi, paraxiphoid thalamic nucleus; pRE, perireuniens; PrS, presubiculum; RE, nucleus reuniens; Rh, rhomboid nucleus; SMT, submedial thalamic nucleus; VL, ventrolateral thalamic nucleus; VM, ventromedial thalamic nucleus; vSUB, ventral subiculum; ZI, zona incerta. Scale bars, 200 μm.

**Figure 7.**
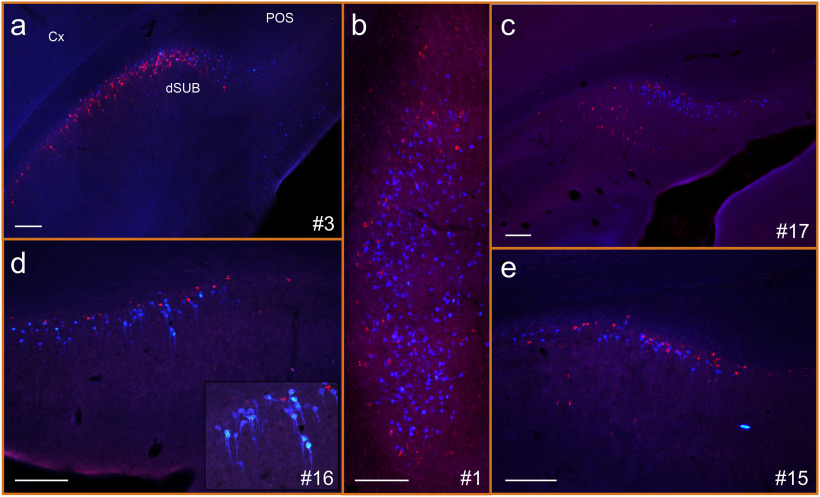
Photomicrographs of retrogradely labeled cells in dorsal and intermediate subiculum in five different cases. Case numbers are indicated in each frame. ***a***, ***b***, Injection sites are restricted to nucleus reuniens and the anterior thalamic nuclei. ***c–e***, Other areas were involved in the injection sites (Table 1). In case #3, the Fast Blue injection (blue cells) is in nucleus reuniens and the CTB injection (red cells) is in the anterior thalamic nuclei. In all other cases, this is reversed. ***a***, Labeled cells resulting from both tracer injections are intermixed in the deep cellular layers. Nevertheless, a tendency for laminar separation is visible with cells projecting to nucleus reuniens (blue) being positioned in the deepest portion. ***b***, At very caudal levels of the intermediate subiculum (where in coronal sections, all cells belong to the deep cell layer), the cells targeting nucleus reuniens (blue) are located in the deepest portion, whereas cells targeting the anterior thalamic nuclei are more equally distributed, but avoid the deepest portion, creating a differentiation across these deepest layers. ***c–e***, Independent of the density of the additional cell label in the superficial cell layer, a sublaminar differentiation is present in the deepest layer. ***c*** and ***e*** also show the more superficial subiculum label observed in a subset of reuniens injection cases. The insert in ***d*** is a 20× zoom image of the same section, showing the morphologic details. Images are adjusted for contrast, brightness, and intensity. Cx, Neocortex; POS, postsubiculum; dSUB, dorsal subiculum. Scale bars, 200 μm.

After ATN injections, the retrograde subiculum cell label was virtually confined to the deep cell portions (Figs. 6, 7), with much of this label in pyramidal cells. While RE injections also resulted in a plexus of deep labeling, this label was often immediately adjacent to the alveus (i.e., even deeper). Consequently, while some labeled cells were intermixed in deep positions, these two pathways were frequently organized in a laminar manner such that the tracer from RE was more deeply placed than the tracer from ATN (Figs. 6*b_6_,c_9_*, 7). The extent of intermixing tended to be greater in cases with injections restricted to AM, while cases including more of AV and AD had less apparent intermingling with RE-labeled cells. Finally, some RE injections had an additional, superficial scattering of subiculum label (Figs. 6, 7), though this was most sparse in the cases with injections confined to RE (cases #1, #2, #3, #5, #9, #14, and #18).

Furthermore, although both cell populations predominantly occupied the deep subiculum, they often differed in morphology. While most cells labeled by ATN injections were pyramidal with apparent apical dendrites extending toward the superficial layers, the cells labeled by RE injections were more variable (polymorphic). This difference was observed in all cases, independent of tracer type (Fig. 7*d*, insert). The lack of a clear, independent division within the subicular cell layers meant that quantitative assessments were not conducted on depth effects.

**Figure 8.**
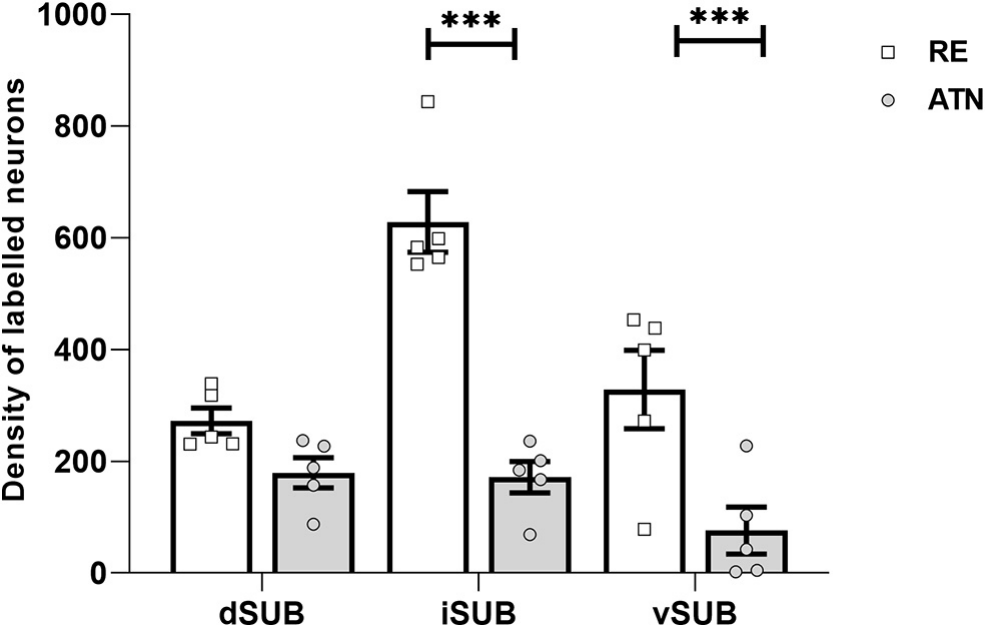
Density of labeled neurons in subiculum (per square millimeter). Plots showing mean density and SEM of retrograde labeled cells in five highlighted cases. Densities are given for the dorsal (dSUB), intermediate (iSUB), and ventral (vSUB) subiculum. ATN, anterior thalamic injections; RE, nucleus reuniens injections. ***p < 0.001.

Tracer injections in the anterior thalamic nuclei appeared to lead to more labeling in dorsal and intermediate subiculum, while the nucleus reuniens label was more evident across all three subiculum divisions, with an apparent peak in the intermediate subiculum (Figs. 6, 8). Matching these impressions, an ANOVA yielded a main effect of injection site (*F*_(1,4)_ = 19.58, *p* = 0.012), no main effect of region (*F*_(2,8)_ = 4.02, *p* = 0.062), and an interaction between injection site and region (*F*_(2,8)_ = 11.61, *p* = 0.004).

A simple main-effects analysis confirmed that there were higher counts after RE injections in the intermediate (*F*_(1,8)_ = 26.28, *p* <0.001) and ventral subiculum relative to after ATN injections (*F*_(1,8)_ = 73.06, *p* < 0.001), but there were no differences in dorsal subiculum (*F*_(1,8)_ = 3.01, *p* = 0.121).

Further analysis also showed a simple main effect of subregion after RE injections (*F*_(2,8)_ = 6.329, *p* = 0.022). *Post hoc* tests showed that there were higher cell counts in intermediate subiculum relative to dorsal subiculum (*p* = 0.001). There was also a simple main effect of region after ATN injections (*F*_(2,8)_ = 28.62, *p* < 0.001), but no further comparisons were significant (minimum *p* = 0.214).

### Mammillary body afferents

All cases with ATN injections resulted in dense cell labeling in the medial mammillary nucleus (Fig. 5*b*). Likewise, the lateral mammillary nucleus was repeatedly labeled in the ATN cases, though with much variation in density between cases. In contrast, in only one of the cases with the tracer injection seemingly restricted to RE (case #5) did we observe moderate retrograde cell labeling in the mammillary bodies, largely confined to the median nucleus. In a few of the other cases with restricted RE injections, we only observed some single scattered cells. No double-labeled cells were observed in the mammillary bodies.

### Cortical and subiculum axon collaterals

#### Double-labeled cell proportions: cortical areas

**Figure 9.**
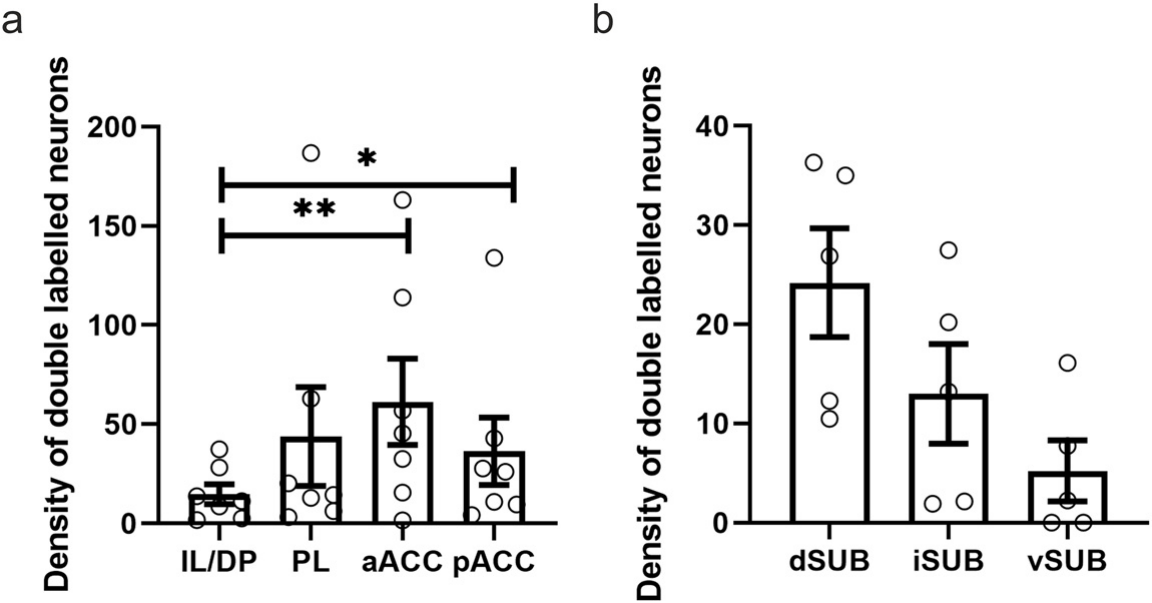
Plots showing the density of double-labeled cells in cortex and subiculum (per square millimeter). ***a***, Plots showing the mean density and SEM of double-labeled cells in infralimbic/dorsal peduncular cortices (IL/DP), prelimbic cortex (PL), rostral anterior cingulate cortex (aACC) and caudal anterior cingulate cortex (pAAC). ***b***, Plots showing the mean density and SEM of double-labeled cells in the dSUB, iSUB, and vSUB. **p < 0.01; *p < 0.05.

**Figure 10.**
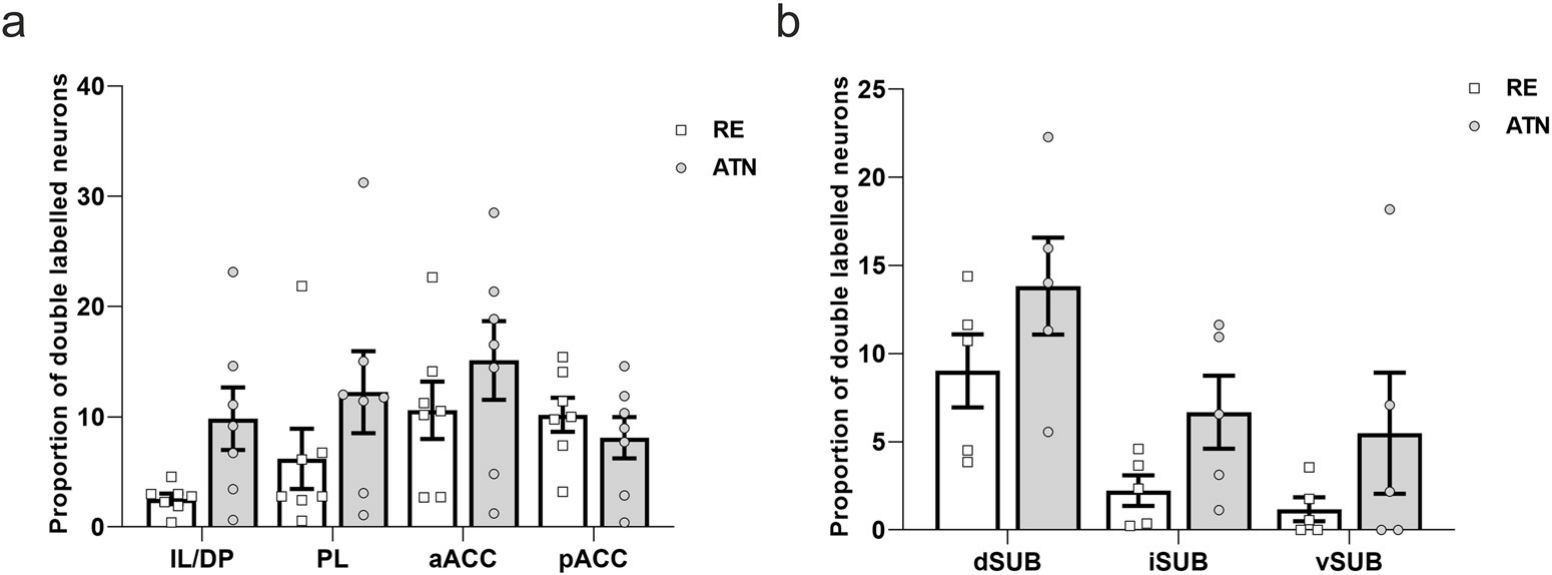
Plots showing the proportions of double-labeled cells in cortex and subiculum. Numbers are given as proportions of cells labeled by the tracer in either the anterior thalamic nuclei (ATN) or nucleus reuniens (RE). ***a***, Plots showing the mean proportions and SEM of double-labeled cells in infralimbic/dorsal peduncular cortices (IL/DP), prelimbic cortex (PL), rostral anterior cingulate cortex (aACC) and caudal anterior cingulate cortex (pAAC). ***b***, Plots showing the mean proportions and SEM of double-labeled cells in the dorsal (dSUB), intermediate (iSUB) and ventral subiculum (vSUB). As these data are not independent, no statistical analyses are shown.

A moderate proportion of the retrogradely labeled cells in cortex were double labeled (Fig. 9*a*). When analyzed as a proportion of the cells labeled by the tracer in ATN, these proportions varied relatively little (Fig. 10*a*). The corresponding proportions for the RE injections showed a greater range, but this, in part, reflects the unequal level of inputs from ventral prefrontal areas to the two thalamic nuclei (Fig. 4*a*), with appreciably less label following ATN injections. These data (Fig. 10*a*) cannot be analyzed statistically as the data are not independent.

Figure 9*a* shows the distribution of double-labeled cells across the four cortical sites. Infralimbic/dorsal peduncular cortices contained the fewest number of double-labeled cells, with no differences between the other cortical sites.

ANOVA confirmed that there were regional differences in the number of double-labeled cells (*F*_(3,18)_ = 4.42 *p* = 0.017). *Post hoc* analysis showed that there were fewer double-labeled cells in infralimbic/dorsal peduncular relative to both the rostral portion of ACC (*p* = 0.009) and the caudal portion of the ACC (*p* = 0.02). No other significant differences were found (minimum *p* = 0.62).

In the retrosplenial cortex (not included in the analysis above), only in one case did we see double-labeled cells (constituting 1.63% of the population of ATN-projecting cells and 22.67% of the RE-projecting cells).

#### Double-labeled cell proportions: subiculum

As was the case for cortical regions, we observed a moderate number of double-labeled cells in the subiculum (Fig. 9*b*). The proportion of double-labeled cells was highest in the dorsal subiculum, and this was the case whether the double-labeled cells were counted as a proportion of the cells labeled by the tracer in RE or the tracer in ATN (9.02% and 13.82%, respectively; Fig. 10*b*)

Across the three subicular subregions, there was an overall difference in the number of double-labeled cells in the three subicular subregions (*F*_(2,8)_ = 6.25, *p* = 0.023). However, *post hoc* tests did not reveal any significant differences among the three subicular subregions (minimum *p* = 0.15).

## Discussion

While the anterior thalamic nuclei and nucleus reuniens are interconnected with many common sites and are involved in overlapping functions, they have different functional attributes (Mathiasen et al., 2020; Cassel et al., 2021). Current comparisons between the afferents to nucleus reuniens and the anterior thalamic nuclei are indirect, relying on separate studies. By placing different retrograde tracers within these two thalamic sites in the same rats, it was possible to compare their inputs directly. Three main issues are considered: (1) whether any general features distinguish the locations of their respective cortical inputs; (2) whether there are laminar differences in the respective cells of origin; and (3) the frequency and location of any neurons that project to both thalamic nuclei.

One challenge in such studies is to achieve sufficient tracer coverage in both target areas. For this reason, most ATN injections were centered in the anteromedial nucleus, which has the greatest array of frontal connections within this nuclear group (Shibata and Naito, 2005; Wright et al., 2013); making it especially informative when drawing comparisons with nucleus reuniens. Given the challenge of restricting injections within nucleus reuniens, prior descriptions of afferents to nucleus reuniens provide valuable checks (Herkenham, 1978; Van der Werf et al., 2002; McKenna and Vertes, 2004; Mathiasen et al., 2019). However, to avoid unwanted uptake in the anterior thalamic nuclei from the syringe tract reaching nucleus reuniens, most RE injections targeted posterior portions of that nucleus. Consequently, a systematic comparison between inputs to anterior and posterior nucleus reuniens was not possible.

One general difference in the cortical afferents to the two thalamic sites concerns their dorsal/ventral and rostral/caudal origins, which were confirmed by quantitative analyses. We repeatedly observed a bias so that, compared with input to nucleus reuniens, relatively more anterior thalamic inputs arrive from more dorsal regions, namely, the anterior cingulate cortex and retrosplenial cortex (but see also van Groen and Wyss, 1990b; Shibata, 1998; Shibata and Naito, 2005; Wright et al., 2013). Likewise, fewer inputs to anterior thalamic nuclei arrive from ventral and intermediate subiculum, again in direct comparison with nucleus reuniens afferents. This pattern also resulted in a rostral–caudal gradient in medial cortical afferents to the anterior thalamic nuclei, with lower cell counts in infralimbic cortex than the posterior anterior cingulate cortex (Fig. 4*a*). The preference for the retrosplenial projections to target the anterior thalamic nuclei (AV in particular) adds to the rostrocaudal topography. At the same time, cortical inputs to nucleus reuniens, while again including inputs from the anterior cingulate cortex, included dense projections from more rostral and ventral areas, namely the prelimbic cortex, infralimbic cortex, and dorsal peduncular cortex (see also Herkenham, 1978; McKenna and Vertes, 2004; Varela et al., 2014), but far fewer from retrosplenial cortex (with, however, significant fewer infralimbic/dorsal peduncular afferents, than from rostral portion of the anterior cingulate). Further support for these gradient differences comes from a transynaptic tracing study showing monosynaptic links from dorsal cortical areas (anterior cingulate and retrosplenial) via the anterior thalamic nuclei to more dorsal hippocampal areas, while ventral prefrontal areas project to more ventral parts of the hippocampal formation via nucleus reuniens (Prasad and Chudasama, 2013).

The dense inputs from retrosplenial cortex and the dorsal subiculum to the anterior thalamic nuclei are presumed to contribute to why this thalamic area is so critical for spatial learning (Nelson et al., 2015, 2020; Ritchey et al., 2015; Yamawaki et al., 2019). In the Morris water maze, for example, rats with anterior thalamic lesions consistently fail to learn the escape location (i.e., they seem unable to distinguish spatial locations; Sutherland and Rodriguez, 1989; Warburton et al., 1999; Wolff et al., 2008). In contrast, rats with nucleus reuniens lesions can initially learn the escape location (Dolleman-van der Weel, 2009; Loureiro et al., 2012), but may show deficits after lengthy retention delays (Loureiro et al., 2012) or when required to switch from response to place-based strategies (Cholvin et al., 2013). These behavioral differences can be linked to the greater inputs to the anterior thalamic nuclei from sites repeatedly implicated in allocentric processing (i.e., the retrosplenial cortex, dorsal hippocampus, and mammillary bodies; Sziklas and Petrides, 1998; Bannerman et al., 1999; Santín et al., 1999; Vann and Aggleton, 2002, 2003; Lukoyanov et al., 2005; Strange et al., 2014). Meanwhile, the less consistent pattern of spatial deficits associated with nucleus reuniens damage reflects its presumed role in regulating medial frontal–hippocampal interactions (Vertes et al., 2007; Cholvin et al., 2013; Griffin, 2015; Viena et al., 2018; Mathiasen et al., 2020).

A second feature that distinguished the cortical projections to the two thalamic sites was the laminar depths of the cells providing their respective inputs. A similar bias was seen in the subiculum, where neurons projecting to nucleus reuniens were typically closer to the alveus. There was a consistent pattern for cortical neurons reaching nucleus reuniens to be principally located in the deepest cortical sublayer (VIb). The specificity of the association between nucleus reuniens projections and VIb was particularly striking in the most ventral frontal portions. Meanwhile, the inputs to the anterior thalamus were more evenly distributed across layers VIa and VIb. An apparent exception was the lateral orbital field where the bilaminar differentiation of layer VI was not visible. We currently do not know the functional significance of these sublaminar differences, but they likely reflect the divergent attributes of these thalamic nuclei. While there is good evidence that nucleus reuniens acts as a hub coordinating medial–hippocampal interactions (Vertes et al., 2007; Mathiasen et al., 2020), the functional importance of anterior thalamic interactions with frontal areas is currently only very poorly understood (Nelson, 2021). Meanwhile, although previous studies do not appear to have reported differential thalamic projections from layer VI sublayers in rat frontal cortices, the somatosensory cortex is thought to project differentially to the thalamus from layer VIa and layer VIb (Bourassa et al., 1995; Killackey and Sherman, 2003; see also Vertes et al., 2015).

The significance of the sublaminar difference in the present study may relate to the heterogeneity of layer VI cells (Tsumoto and Suda, 1980; Sirota et al., 2005; Briggs, 2010). One example concerns the relative distributions of “short” versus “tall” pyramidal neurons across layers VIa and VIb (Lund et al., 1979; Briggs, 2010), which may then differentially innervate the two thalamic nuclei under investigation. Another example concerns the intensity of parvalbumin expression, which helped to differentiate layer VIa and layer VIb in the ventral prefrontal and anterior cingulate cortices (but also see Paxinos et al., 1999). While parvalbumin expression also differentiated deep from superficial cell layers in the dorsal peduncular cortex, this same staining difference was not seen in the lateral orbital field, where the populations of cells projecting the two thalamic nuclei were the most intermixed.

Other authors have used a variety of nomenclatures for the sublaminar differentiation of layer VI, but the cytoarchitectonic criteria appear common across studies. For instance, in cortical areas lateral to the anterior cingulate cortex, cells deep in the intervening cell-sparse zone have been termed either layer VIb (Kristt, 1979; Valverde et al., 1989; Killackey and Sherman, 2003) or layer VII (alternatively, the “subgriseal layer”; Reep and Goodwin, 1988; Clancy and Cauller, 1999). These terms refer to the same laminar portions, but with a residual inconsistency over whether the cell-sparse zone is included. In medial prefrontal cortex, Vogt and Paxinos (2014) identified three sublayers of layer VI, termed a, b, and c. Layer VIb in the present study corresponds to the combined sublayers b and c. Likewise, Jones et al. (2005) differentiated layers VIa and VIb but only recognized this bilaminar organization in the dorsal anterior cingulate cortex (i.e., excluding both ventral anterior cingulate and the remaining frontal cortices). Interestingly, the same study (Jones et al., 2005) also stained for parvalbumin but did not report a bilaminar differentiation in ventral portions of the frontal cortex. However, as layer VIa progressively thins in more ventral prefrontal levels, it is possible that layer VIa in this, and potentially other studies, has been included in layer V.

Most subiculum cells projecting to nucleus reuniens and the anterior thalamic nuclei were located in its deep cellular layer (perialvear), though, once again, the anterior thalamic inputs were typically positioned slightly superficial to those neurons reaching nucleus reuniens. Although the cellular layer of the rat subiculum can appear fairly homogeneous (Kloosterman et al., 2003), it contains nonpyramidal cells adjacent to the alveus (Ishihara and Fukuda, 2016) that may form part of an additional layer (Ishizuka, 2001). Indeed, the cells projecting to nucleus reuniens often appeared polymorphic (i.e., nonpyramidal), while those innervating the anterior thalamic nuclei were more frequently pyramidal. Gene expression analyses also highlight lamination deep within the rodent subiculum (Bienkowski et al., 2018). Other depth differences include evidence that deep subiculum pyramidal cell neurons are more likely to show intrinsic bursting, as well as having longer apical dendrites, but more restricted collateralization, than more superficial pyramidal cells in the subiculum (Harris et al., 2001). These deep bursting neurons potentially contribute to the anterior thalamic inputs, which arise from pyramidal cells in this layer.

It has been reported that nucleus reuniens receives inputs not only from deep subiculum cells but also from pyramidal cells at appreciably more superficial levels within the subiculum (Herkenham, 1978; McKenna and Vertes, 2004), providing it with a more heterogeneous hippocampal input. In the present study, these more superficial subiculum cells, although present, were only evident in substantial numbers, when the tracer injection spread beyond nucleus reuniens. The implication, that nucleus reuniens itself is principally innervated by very deep (perialvear) subiculum cells, is supported by two recent mouse studies (Bienkowski et al., 2018; Scheel et al., 2020; see also Mathiasen et al., 2019).

The only subcortical nuclei to be studied in detail in the present study were the mammillary bodies, given the density of their projections to the anterior thalamic nuclei (Seki and Zyo, 1984; Shibata, 1992). A much lighter projection from the mammillary bodies to nucleus reuniens has been previously described (McKenna and Vertes, 2004). In the present study, only one case contained a substantial retrograde label in the mammillary bodies following an injection targeting nucleus reuniens, which was localized but relatively dense. Consequently, our results indicate that any mammillary body inputs to the rat nucleus reuniens are typically either extremely sparse or topographically confined.

Beyond the mammillary bodies, the anterior thalamic nuclei receive subcortical inputs from a very restricted number of subcortical sites. These additional subcortical sites include the thalamic reticular nucleus (Gonzalo-Ruiz and Lieberman, 1995; Lozsadi, 1995), the laterodorsal tegmental nucleus (Cornwall et al., 1990; Gonzalo-Ruiz et al., 1995b), the pedunculopontine tegmental nucleus, and the median raphe nucleus (Vertes, 1991; Gonzalo-Ruiz et al., 1995a,b; Vertes et al., 1999). It is striking that nucleus reuniens not only receives subcortical afferents from these same sites, but also receives additional projections from a wide array of other subcortical nuclei (McKenna and Vertes, 2004). Consequently, the breadth of their subcortical inputs provides a clear difference between the operations of the anterior thalamic nuclei and nucleus reuniens.

A third goal was to assess the prevalence of individual cortical neurons that project to both thalamic nuclei. All frontal cortical sites examined contained some double-labeled cells, indicative of neurons that innervate both thalamic nuclei. As might be expected, these cells were most frequent in the zones of overlap between the sublaminar levels in layer VI. A similar pattern was observed in the deepest layer of the subiculum. The overall numbers of double-labeled cells highlight how they consistently comprised a modest minority of afferents, with evidence of a slight increase in the anterior cingulate cortices and dorsal subiculum (∼10–15%). This percentage is more likely to be an underestimate rather than an overestimate, given the challenge of filling both thalamic target sites with tracer. Furthermore, when considering the relative proportions of double-labeled cells, it must be remembered that the thalamic nucleus receiving the fewer number of inputs will always have the higher proportion of double-labeled cells (as the number of double-labeled cells must remain fixed). For this reason, proportional counts must be interpreted with caution. Finally, in no case could double-labeled cells be found in the mammillary bodies.

The minority of double-labeled cells, combined with the bias for projections to nucleus reuniens and the anterior thalamic nuclei to originate from overlapping, but different, depths would indicate that these two thalamic sites receive closely related information, which, in the main, is separated. To this pattern of potential segregation can be added previous evidence of an apparent lack of individual cells that project to both the mammillary bodies and nucleus reuniens (Mathiasen et al., 2019), as well as a lack of individual neurons that innervate more than one anterior thalamic nucleus (Wright et al., 2013) or innervate both the anterior thalamic nuclei and the mediodorsal thalamic nucleus (Wright et al., 2013). Their presence, albeit in a minority, does highlight how the anterior thalamic nuclei and nucleus reuniens operate in a complementary manner in common domains.

In summary, the present study reveals pervasive differences between the inputs to nucleus reuniens and the anterior thalamic nuclei, although almost every brain site that innervates the anterior thalamic nuclei also appears to project to nucleus reuniens, with the potential exception of the mammillary bodies. The following three key afferent differences were observed: (1) their inputs show differential dorsal/ventral and rostral/caudal cortical/allocortical gradients; (2) their cortical inputs frequently occupy different laminar position within layer VI; and (3) that, even when intermingled, only a modest proportion of neurons project to both sites. All three differences highlight ways in which these two thalamic sites have distinct functional roles, while at the same time reinforcing the notion that they operate in overlapping cognitive domains.

## Synthesis

Reviewing Editor: Thomas McHugh, RIKEN Center for Brain Science Decisions are customarily a result of the Reviewing Editor and the peer reviewers coming together and discussing their recommendations until a consensus is reached. When revisions are invited, a fact-based synthesis statement explaining their decision and outlining what is needed to prepare a revision will be listed below. The following reviewer(s) agreed to reveal their identity: L. Ian Schmitt.

Summary and Synthesis

Despite the increasing availability of systematic anterograde tracing data to assess connections among brain regions, direct comparison of the inputs to functionally related circuits using multi-site tracing remains an important to reveal networks that underlie cognitive processes. This study applies retrograde tracing methods to investigate the inputs to two regions of the thalamus: the nucleus reunions and anterior thalamic nuclei. Both ATN and RE are densely connected with the prefrontal cortex (PFC) and hippocampus, and are involved in learning and memory. Although the connectivity of these regions has been well described in previous anatomical studies, because of the similarity of inputs and because these data were achieved from separate studies, the detailed differences in afferents were unclear. In this study, the authors directly compared the differences of afferents to the ATN and RE by conducting dual retrograde tracing in rats. The results show that the deepest sublayer (VIb) of medial prefrontal cortex target RE whereas the projection to ATN originates from more superficial sublayers (VIa). Similar laminar differences were also observed in the projection from Subiculum to ATN and RE. In addition to these laminar differences, the differences of the input regions between ATN and RE are shown along the rostro-caudal axis. These interesting anatomical findings may contribute to understand the neural basis which underlie the comparative roles of these thalamic nuclei in learning and memory.

While both referees find the revised manuscript to be much improved, noting the interesting and useful new insights and further commenting that the additional samples which were added in this revised manuscript are beautiful and support their claims, they do have some remaining suggestions to improve the quality and readability of the paper. Three major classes of concerns, along with two minor suggestions are detailed below.

1. Issues regarding quantification and data.

a. In the additional quantification data, the authors show the “the cell count” by dividing “the total number of labelled cells” by “the number of counted sections”. This may not be accurate since each brain region differ in the area size and the number of sections which they are included. For example, a region which extend along the dosoventral axis will likely have a higher number than a region which extend along the caudorostral axis. This may be a reason why iSub, which extend along the dorsobentral axis (Figure 6b and Figure 8b), show higher number than dSub and vSub. To make the quantification accurate in Figure 4a and Figure 7, “the total number of labelled cells” should be divided by “the area size of the brain region” and show the density of the labelled cells.

b. As shown in Figure 3e, aRSC projects to both RE and AM. To support the claim that the projections to the two thalamic nucleus are organized in opposing rostral/caudal gradients in the cortex, the quantification for RSC should also be included in Figure 4a.

c. The data for IL/DP (and preferably RSC) should be included in Figure 4b.

d. The percentage of the double-labeled cells are important, but it is currently shown only in the table which the value in each sample is unclear. Rather than showing them in a table, it would be informative to show them as a graph in Figure 9.

2. Issues related to manuscript organization and data presentation

a. In the results for “Distribution of cortical projections (Figure 3-4)” and “Hippocampal formation: Laminar... (Figure 6-7)” the additional quantifications are first described followed by the explanation of the representative case. This organization is difficult to read. It would be better to describe along the order of the figures: first explain the labeling patterns by showing the representative data, and then the quantitative data to show consistency and robustness of the results.

b. Though important, figure 1 is complex and its organizational scheme difficult to identify. Some additional labelling and reorganization would be beneficial.

c. Figure 2 appears disorganized and is hard to follow visually. It also does not follow the conventions of labelling set in figure 1 (i.e. overlaying the numbers of the sample onto the image within the orange box). The horizontal line in the center of the figure separating the #9 and #3 group is not a clear way to demarcate these samples. In figure 2j-m, the border of the layers should be shown not with arrows but with dotted lines. This is because we cannot see how the border extends only with the arrows.

d. Case numbers are not consistently labelled (e.g. they are identified in figure 2 but not in figure 5). Either way is fine but it should be consistent. Also the images are not outlined in figure 5 in orange as they are in other figures. In figure 5a the border should be shown with dotted lines, and layer Va and Vb should be dissociated. It is also impossible to know which AP level this section was taken from. The low magnification image or schematic drawing of the section should be shown to identify the overall location of where the image was taken from.

e. In figure 6, it is not apparent at a first glance where “b4-6” was taken from. This should be improved. Additionally, in figure 6, the double-labeled cells are highlighted by the white arrows only in the merged image (b3). Arrows should also be shown in (b1) and (b2). In addition, there also seemed to be double-labeled neurons in (c1-3), (c4-6), and (c7-9). These also should be highlighted.

3. Issues related to the discussion.

a. The connectivity differences between layer VIa and VIb neurons is interesting but the potential functional meaning is unclear in the discussion. Since this is one of their main findings, the functional implication should be discussed.

Minor points

1. Though much clarified and improved, there are still some issues with the writing, particularly in the figure legends. For example, the term “Histogram” is incorrectly applied to describe bar/dot plots in several figures. The overall document should be gone through carefully to avoid such issues with terminology.

2. In sample #5, the blue tracer (FB?) is injected in RE while the red tracer (CTB?) is injected in AV (Figure 1). In the table, however, it says that CTB is injected in RE whereas FB is injected in AV. Please check and resolve the discrepancy.
